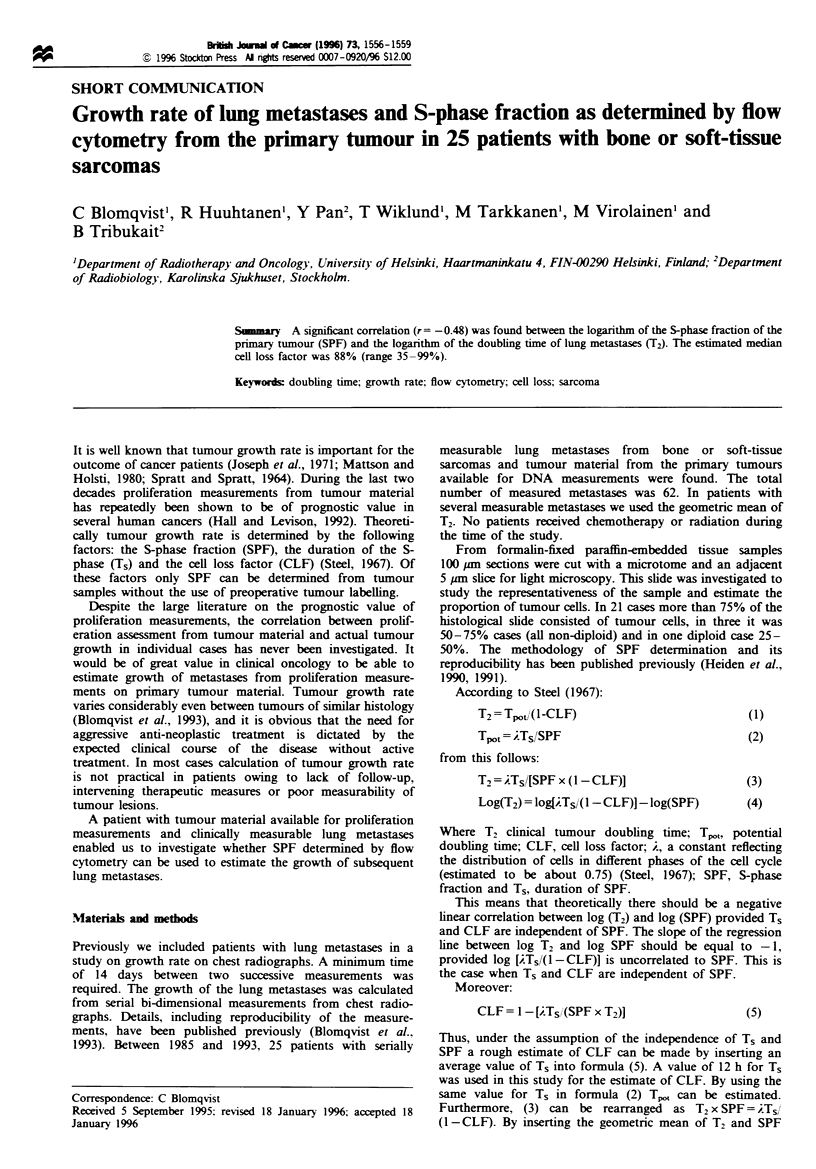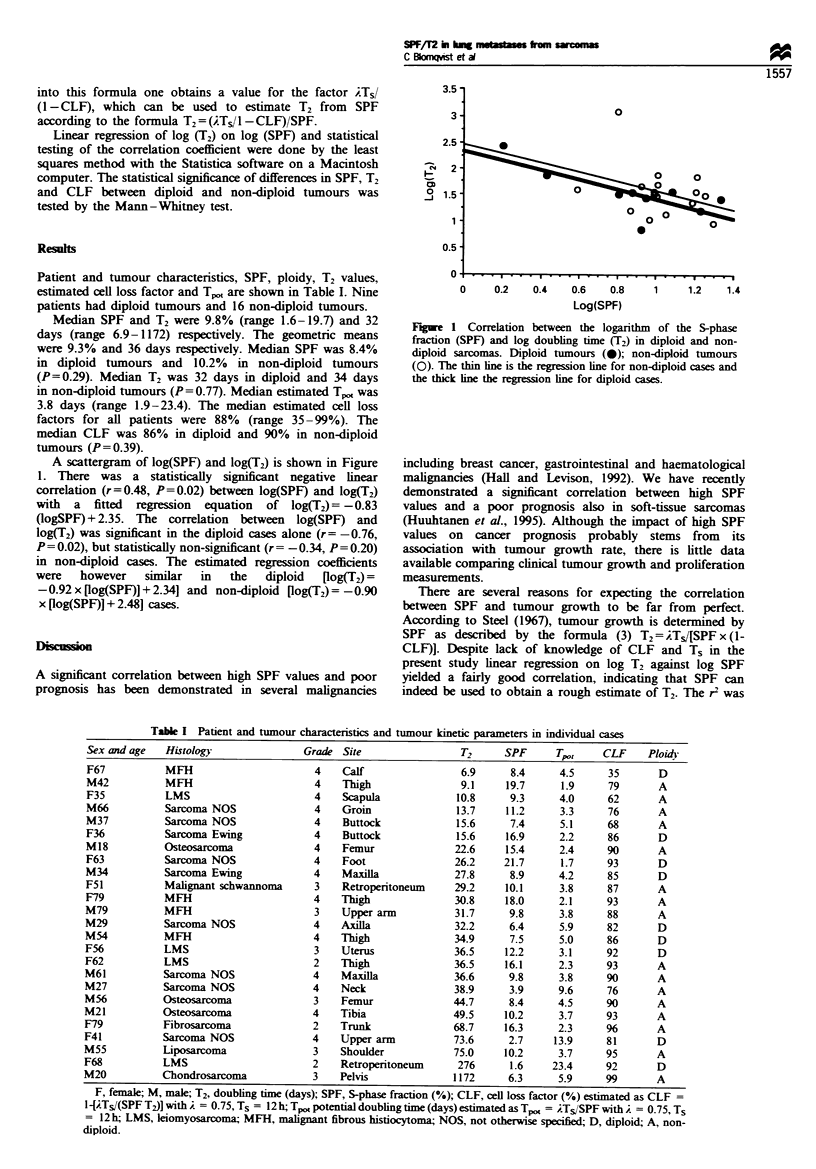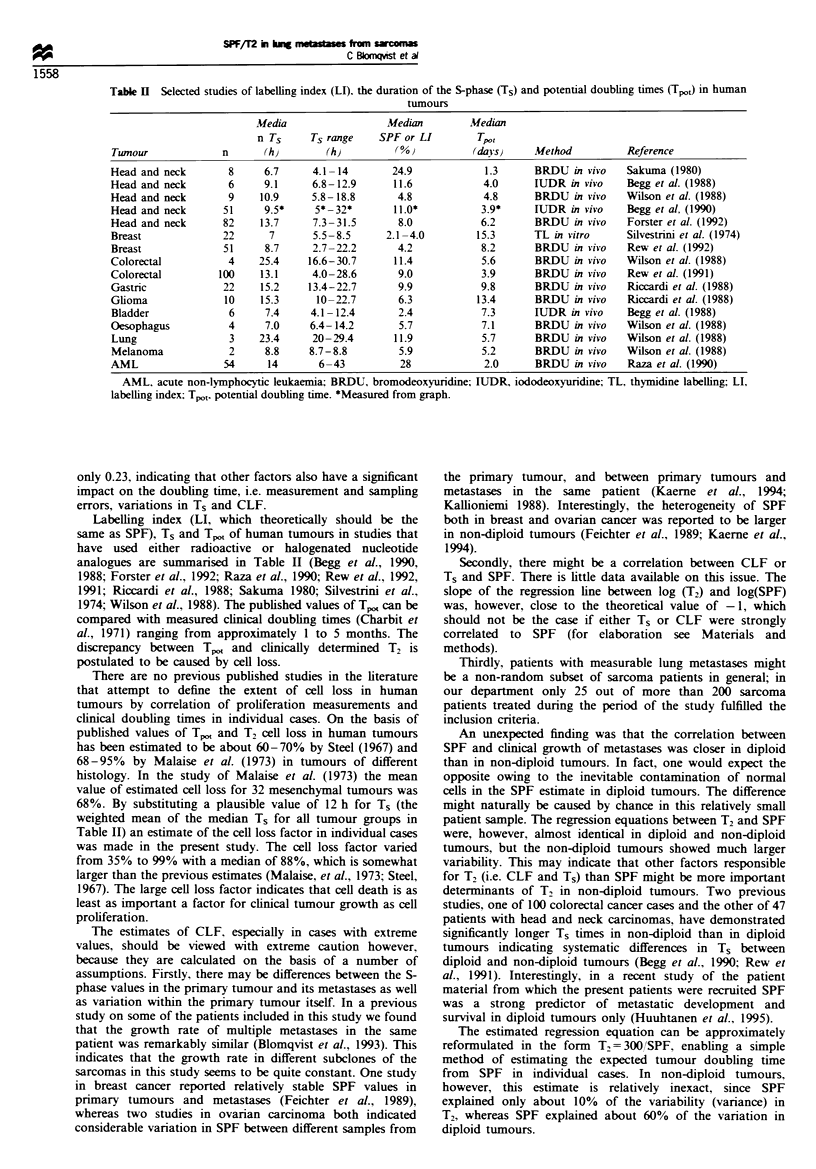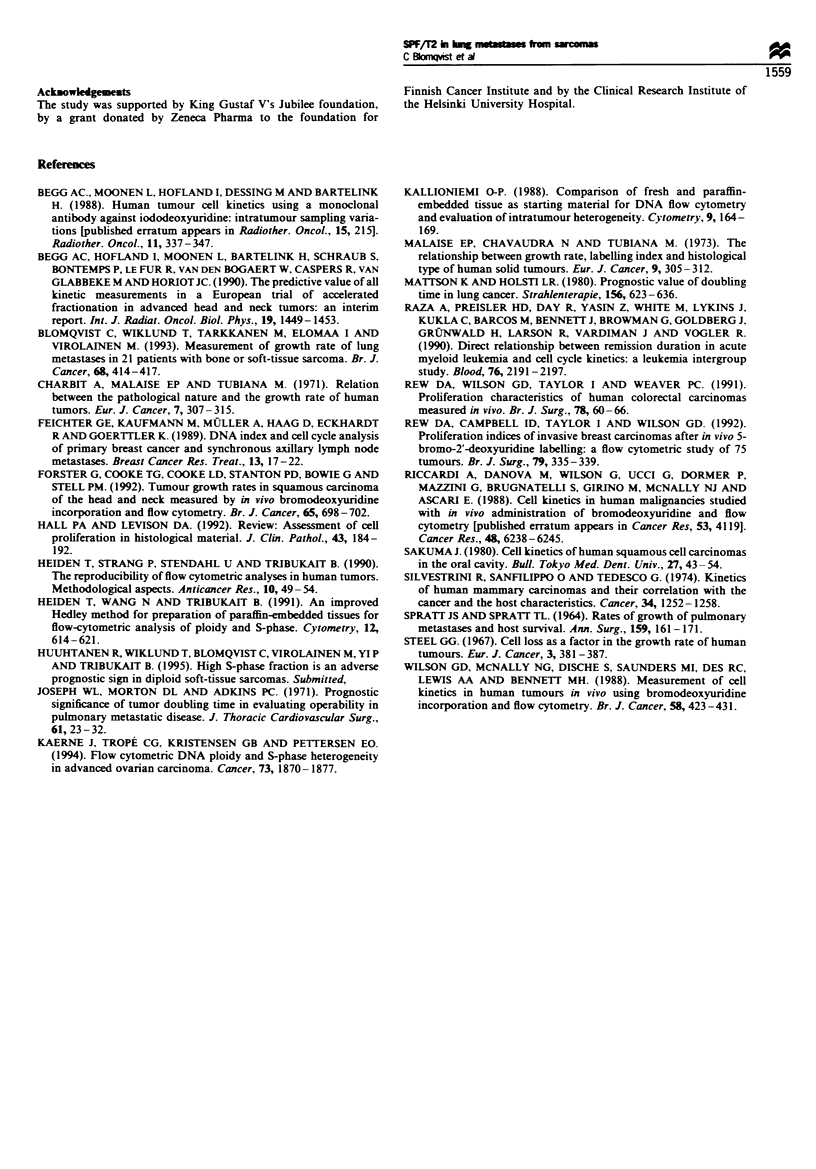# Growth rate of lung metastases and S-phase fraction as determined by flow cytometry from the primary tumour in 25 patients with bone or soft-tissue sarcomas.

**DOI:** 10.1038/bjc.1996.293

**Published:** 1996-06

**Authors:** C. Blomqvist, R. Huuhtanen, Y. Pan, T. Wiklund, M. Tarkkanen, M. Virolainen, B. Tribukait

**Affiliations:** Department of Radiotherapy and Oncology, University of Helsinki, Finland.

## Abstract

A significant correlation (r = -0.48) was found between the logarithm of the S-phase fraction of the primary tumour (SPF) and the logarithm of the doubling time of lung metastases (T2). The estimated median cell loss factor was 88% (range 35-99%).


British Journal of Cancer (1996) 73, 1556-1559

© 1996 Stockton Press All rights reserved 0007-0920/96 $12.00

SHORT COMMUNICATION

### Growth rate of lung metastases and S-phase fraction as determined by flow cytometry from the primary tumour in 25 patients with bone or soft-tissue sarcomas

C Blomqvist[1], R Huuhtanen[1], Y Pan[2], T Wiklund[1], M Tarkkanen[1], M Virolainen[1] and B Tribukait[2]

[1]*Department of Radiotherapy and Oncology, University of Helsinki, Haartmaninkatu 4, FIN-00290 Helsinki, Finland;* [2]*Department of Radiobiology, Karolinska Sjukhuset, Stockholm.*

**Summary** A significant correlation ($r = -0.48$) was found between the logarithm of the S-phase fraction of the primary tumour (SPF) and the logarithm of the doubling time of lung metastases ($T_2$). The estimated median cell loss factor was 88% (range 35–99%).

**Keywords:** doubling time; growth rate; flow cytometry; cell loss; sarcoma

It is well known that tumour growth rate is important for the outcome of cancer patients (Joseph *et al.*, 1971; Mattson and Holsti, 1980; Spratt and Spratt, 1964). During the last two decades proliferation measurements from tumour material has repeatedly been shown to be of prognostic value in several human cancers (Hall and Levison, 1992). Theoretically tumour growth rate is determined by the following factors: the S-phase fraction (SPF), the duration of the S-phase ($T_S$) and the cell loss factor (CLF) (Steel, 1967). Of these factors only SPF can be determined from tumour samples without the use of preoperative tumour labelling.

Despite the large literature on the prognostic value of proliferation measurements, the correlation between proliferation assessment from tumour material and actual tumour growth in individual cases has never been investigated. It would be of great value in clinical oncology to be able to estimate growth of metastases from proliferation measurements on primary tumour material. Tumour growth rate varies considerably even between tumours of similar histology (Blomqvist *et al.*, 1993), and it is obvious that the need for aggressive anti-neoplastic treatment is dictated by the expected clinical course of the disease without active treatment. In most cases calculation of tumour growth rate is not practical in patients owing to lack of follow-up, intervening therapeutic measures or poor measurability of tumour lesions.

A patient with tumour material available for proliferation measurements and clinically measurable lung metastases enabled us to investigate whether SPF determined by flow cytometry can be used to estimate the growth of subsequent lung metastases.

### Materials and methods

Previously we included patients with lung metastases in a study on growth rate on chest radiographs. A minimum time of 14 days between two successive measurements was required. The growth of the lung metastases was calculated from serial bi-dimensional measurements from chest radiographs. Details, including reproducibility of the measurements, have been published previously (Blomqvist *et al.*, 1993). Between 1985 and 1993, 25 patients with serially measurable lung metastases from bone or soft-tissue sarcomas and tumour material from the primary tumours available for DNA measurements were found. The total number of measured metastases was 62. In patients with several measurable metastases we used the geometric mean of $T_2$. No patients received chemotherapy or radiation during the time of the study.

From formalin-fixed paraffin-embedded tissue samples 100 $\mu m$ sections were cut with a microtome and an adjacent 5 $\mu m$ slice for light microscopy. This slide was investigated to study the representativeness of the sample and estimate the proportion of tumour cells. In 21 cases more than 75% of the histological slide consisted of tumour cells, in three it was 50–75% cases (all non-diploid) and in one diploid case 25–50%. The methodology of SPF determination and its reproducibility has been published previously (Heiden *et al.*, 1990, 1991).

According to Steel (1967):

$$T_2 = T_{pot}/(1\text{-CLF}) \quad (1)$$

$$T_{pot} = \lambda T_S/\text{SPF} \quad (2)$$

from this follows:

$$T_2 = \lambda T_S/[\text{SPF} \times (1 - \text{CLF})] \quad (3)$$

$$\text{Log}(T_2) = \log[\lambda T_S/(1 - \text{CLF})] - \log(\text{SPF}) \quad (4)$$

Where $T_2$ clinical tumour doubling time; $T_{pot}$, potential doubling time; CLF, cell loss factor; $\lambda$, a constant reflecting the distribution of cells in different phases of the cell cycle (estimated to be about 0.75) (Steel, 1967); SPF, S-phase fraction and $T_S$, duration of SPF.

This means that theoretically there should be a negative linear correlation between log ($T_2$) and log (SPF) provided $T_S$ and CLF are independent of SPF. The slope of the regression line between log $T_2$ and log SPF should be equal to $-1$, provided log $[\lambda T_S/(1 - \text{CLF})]$ is uncorrelated to SPF. This is the case when $T_S$ and CLF are independent of SPF.

Moreover:

$$\text{CLF} = 1 - [\lambda T_S/(\text{SPF} \times T_2)] \quad (5)$$

Thus, under the assumption of the independence of $T_S$ and SPF a rough estimate of CLF can be made by inserting an average value of $T_S$ into formula (5). A value of 12 h for $T_S$ was used in this study for the estimate of CLF. By using the same value for $T_S$ in formula (2) $T_{pot}$ can be estimated. Furthermore, (3) can be rearranged as $T_2 \times \text{SPF} = \lambda T_S/(1 - \text{CLF})$. By inserting the geometric mean of $T_2$ and SPF

Correspondence: C Blomqvist
Received 5 September 1995; revised 18 January 1996; accepted 18 January 1996

into this formula one obtains a value for the factor $\lambda T_S/(1-CLF)$, which can be used to estimate $T_2$ from SPF according to the formula $T_2=(\lambda T_S/1-CLF)/SPF$.

Linear regression of log ($T_2$) on log (SPF) and statistical testing of the correlation coefficient were done by the least squares method with the Statistica software on a Macintosh computer. The statistical significance of differences in SPF, $T_2$ and CLF between diploid and non-diploid tumours was tested by the Mann-Whitney test.

### Results

Patient and tumour characteristics, SPF, ploidy, $T_2$ values, estimated cell loss factor and $T_{pot}$ are shown in Table I. Nine patients had diploid tumours and 16 non-diploid tumours.

Median SPF and $T_2$ were 9.8% (range 1.6-19.7) and 32 days (range 6.9-1172) respectively. The geometric means were 9.3% and 36 days respectively. Median SPF was 8.4% in diploid tumours and 10.2% in non-diploid tumours ($P=0.29$). Median $T_2$ was 32 days in diploid and 34 days in non-diploid tumours ($P=0.77$). Median estimated $T_{pot}$ was 3.8 days (range 1.9-23.4). The median estimated cell loss factors for all patients were 88% (range 35-99%). The median CLF was 86% in diploid and 90% in non-diploid tumours ($P=0.39$).

A scattergram of log(SPF) and log($T_2$) is shown in Figure 1. There was a statistically significant negative linear correlation ($r=0.48$, $P=0.02$) between log(SPF) and log($T_2$) with a fitted regression equation of $\log(T_2)=-0.83$ (logSPF) + 2.35. The correlation between log(SPF) and log($T_2$) was significant in the diploid cases alone ($r=-0.76$, $P=0.02$), but statistically non-significant ($r=-0.34$, $P=0.20$) in non-diploid cases. The estimated regression coefficients were however similar in the diploid [$\log(T_2)=-0.92\times[\log(SPF)]+2.34$] and non-diploid [$\log(T_2)=-0.90\times[\log(SPF)]+2.48$] cases.

### Discussion

A significant correlation between high SPF values and poor prognosis has been demonstrated in several malignancies including breast cancer, gastrointestinal and haematological malignancies (Hall and Levison, 1992). We have recently demonstrated a significant correlation between high SPF values and a poor prognosis also in soft-tissue sarcomas (Huuhtanen *et al*., 1995). Although the impact of high SPF values on cancer prognosis probably stems from its association with tumour growth rate, there is little data available comparing clinical tumour growth and proliferation measurements.

There are several reasons for expecting the correlation between SPF and tumour growth to be far from perfect. According to Steel (1967), tumour growth is determined by SPF as described by the formula (3) $T_2=\lambda T_S/[SPF\times(1-CLF)]$. Despite lack of knowledge of CLF and $T_S$ in the present study linear regression on log $T_2$ against log SPF yielded a fairly good correlation, indicating that SPF can indeed be used to obtain a rough estimate of $T_2$. The $r^2$ was

**Figure 1** Correlation between the logarithm of the S-phase fraction (SPF) and log doubling time ($T_2$) in diploid and non-diploid sarcomas. Diploid tumours (●); non-diploid tumours (○). The thin line is the regression line for non-diploid cases and the thick line the regression line for diploid cases.

**Table I** Patient and tumour characteristics and tumour kinetic parameters in individual cases

| *Sex and age* | *Histology* | *Grade* | *Site* | $T_2$ | *SPF* | $T_{pot}$ | *CLF* | *Ploidy* |
|---|---|---|---|---|---|---|---|---|
| F67 | MFH | 4 | Calf | 6.9 | 8.4 | 4.5 | 35 | D |
| M42 | MFH | 4 | Thigh | 9.1 | 19.7 | 1.9 | 79 | A |
| F35 | LMS | 4 | Scapula | 10.8 | 9.3 | 4.0 | 62 | A |
| M66 | Sarcoma NOS | 4 | Groin | 13.7 | 11.2 | 3.3 | 76 | A |
| M37 | Sarcoma NOS | 4 | Buttock | 15.6 | 7.4 | 5.1 | 68 | A |
| F36 | Sarcoma Ewing | 4 | Buttock | 15.6 | 16.9 | 2.2 | 86 | D |
| M18 | Osteosarcoma | 4 | Femur | 22.6 | 15.4 | 2.4 | 90 | A |
| F63 | Sarcoma NOS | 4 | Foot | 26.2 | 21.7 | 1.7 | 93 | D |
| M34 | Sarcoma Ewing | 4 | Maxilla | 27.8 | 8.9 | 4.2 | 85 | D |
| F51 | Malignant schwannoma | 3 | Retroperitoneum | 29.2 | 10.1 | 3.8 | 87 | A |
| F79 | MFH | 4 | Thigh | 30.8 | 18.0 | 2.1 | 93 | A |
| M79 | MFH | 3 | Upper arm | 31.7 | 9.8 | 3.8 | 88 | A |
| M29 | Sarcoma NOS | 4 | Axilla | 32.2 | 6.4 | 5.9 | 82 | D |
| M54 | MFH | 4 | Thigh | 34.9 | 7.5 | 5.0 | 86 | D |
| F56 | LMS | 3 | Uterus | 36.5 | 12.2 | 3.1 | 92 | D |
| F62 | LMS | 2 | Thigh | 36.5 | 16.1 | 2.3 | 93 | A |
| M61 | Sarcoma NOS | 4 | Maxilla | 36.6 | 9.8 | 3.8 | 90 | A |
| M27 | Sarcoma NOS | 4 | Neck | 38.9 | 3.9 | 9.6 | 76 | A |
| M56 | Osteosarcoma | 3 | Femur | 44.7 | 8.4 | 4.5 | 90 | A |
| M21 | Osteosarcoma | 4 | Tibia | 49.5 | 10.2 | 3.7 | 93 | A |
| F79 | Fibrosarcoma | 2 | Trunk | 68.7 | 16.3 | 2.3 | 96 | A |
| F41 | Sarcoma NOS | 4 | Upper arm | 73.6 | 2.7 | 13.9 | 81 | D |
| M55 | Liposarcoma | 3 | Shoulder | 75.0 | 10.2 | 3.7 | 95 | A |
| F68 | LMS | 2 | Retroperitoneum | 276 | 1.6 | 23.4 | 92 | D |
| M20 | Chondrosarcoma | 3 | Pelvis | 1172 | 6.3 | 5.9 | 99 | A |

F, female; M, male; $T_2$, doubling time (days); SPF, S-phase fraction (%); CLF, cell loss factor (%) estimated as $CLF = 1-[\lambda T_S/(SPF\ T_2)]$ with $\lambda = 0.75$, $T_S = 12$ h; $T_{pot}$ potential doubling time (days) estimated as $T_{pot} = \lambda T_S/SPF$ with $\lambda = 0.75$, $T_S = 12$ h; LMS, leiomyosarcoma; MFH, malignant fibrous histiocytoma; NOS, not otherwise specified; D, diploid; A, non-diploid.

**Table II** Selected studies of labelling index (LI), the duration of the S-phase ($T_S$) and potential doubling times ($T_{pot}$) in human tumours

| Tumour | n | Median $T_S$ (h) | $T_S$ range (h) | Median SPF or LI (%) | Median $T_{pot}$ (days) | Method | Reference |
|---|---|---|---|---|---|---|---|
| Head and neck | 8 | 6.7 | 4.1-14 | 24.9 | 1.3 | BRDU *in vivo* | Sakuma (1980) |
| Head and neck | 6 | 9.1 | 6.8-12.9 | 11.6 | 4.0 | IUDR *in vivo* | Begg *et al.* (1988) |
| Head and neck | 9 | 10.9 | 5.8-18.8 | 4.8 | 4.8 | BRDU *in vivo* | Wilson *et al.* (1988) |
| Head and neck | 51 | 9.5* | 5*-32* | 11.0* | 3.9* | IUDR *in vivo* | Begg *et al.* (1990) |
| Head and neck | 82 | 13.7 | 7.3-31.5 | 8.0 | 6.2 | BRDU *in vivo* | Forster *et al.* (1992) |
| Breast | 22 | 7 | 5.5-8.5 | 2.1-4.0 | 15.3 | TL *in vitro* | Silvestrini *et al.* (1974) |
| Breast | 51 | 8.7 | 2.7-22.2 | 4.2 | 8.2 | BRDU *in vivo* | Rew *et al.* (1992) |
| Colorectal | 4 | 25.4 | 16.6-30.7 | 11.4 | 5.6 | BRDU *in vivo* | Wilson *et al.* (1988) |
| Colorectal | 100 | 13.1 | 4.0-28.6 | 9.0 | 3.9 | BRDU *in vivo* | Rew *et al.* (1991) |
| Gastric | 22 | 15.2 | 13.4-22.7 | 9.9 | 9.8 | BRDU *in vivo* | Riccardi *et al.* (1988) |
| Glioma | 10 | 15.3 | 10-22.7 | 6.3 | 13.4 | BRDU *in vivo* | Riccardi *et al.* (1988) |
| Bladder | 6 | 7.4 | 4.1-12.4 | 2.4 | 7.3 | IUDR *in vivo* | Begg *et al.* (1988) |
| Oesophagus | 4 | 7.0 | 6.4-14.2 | 5.7 | 7.1 | BRDU *in vivo* | Wilson *et al.* (1988) |
| Lung | 3 | 23.4 | 20-29.4 | 11.9 | 5.7 | BRDU *in vivo* | Wilson *et al.* (1988) |
| Melanoma | 2 | 8.8 | 8.7-8.8 | 5.9 | 5.2 | BRDU *in vivo* | Wilson *et al.* (1988) |
| AML | 54 | 14 | 6-43 | 28 | 2.0 | BRDU *in vivo* | Raza *et al.* (1990) |

AML, acute non-lymphocytic leukaemia; BRDU, bromodeoxyuridine; IUDR, iododeoxyuridine; TL, thymidine labelling; LI, labelling index; $T_{pot}$, potential doubling time. *Measured from graph.

only 0.23, indicating that other factors also have a significant impact on the doubling time, i.e. measurement and sampling errors, variations in $T_S$ and CLF.

Labelling index (LI, which theoretically should be the same as SPF), $T_S$ and $T_{pot}$ of human tumours in studies that have used either radioactive or halogenated nucleotide analogues are summarised in Table II (Begg *et al.*, 1990, 1988; Forster *et al.*, 1992; Raza *et al.*, 1990; Rew *et al.*, 1992, 1991; Riccardi *et al.*, 1988; Sakuma 1980; Silvestrini *et al.*, 1974; Wilson *et al.*, 1988). The published values of $T_{pot}$ can be compared with measured clinical doubling times (Charbit *et al.*, 1971) ranging from approximately 1 to 5 months. The discrepancy between $T_{pot}$ and clinically determined $T_2$ is postulated to be caused by cell loss.

There are no previous published studies in the literature that attempt to define the extent of cell loss in human tumours by correlation of proliferation measurements and clinical doubling times in individual cases. On the basis of published values of $T_{pot}$ and $T_2$ cell loss in human tumours has been estimated to be about 60-70% by Steel (1967) and 68-95% by Malaise *et al.* (1973) in tumours of different histology. In the study of Malaise *et al.* (1973) the mean value of estimated cell loss for 32 mesenchymal tumours was 68%. By substituting a plausible value of 12 h for $T_S$ (the weighted mean of the median $T_S$ for all tumour groups in Table II) an estimate of the cell loss factor in individual cases was made in the present study. The cell loss factor varied from 35% to 99% with a median of 88%, which is somewhat larger than the previous estimates (Malaise, *et al.*, 1973; Steel, 1967). The large cell loss factor indicates that cell death is as least as important a factor for clinical tumour growth as cell proliferation.

The estimates of CLF, especially in cases with extreme values, should be viewed with extreme caution however, because they are calculated on the basis of a number of assumptions. Firstly, there may be differences between the S-phase values in the primary tumour and its metastases as well as variation within the primary tumour itself. In a previous study on some of the patients included in this study we found that the growth rate of multiple metastases in the same patient was remarkably similar (Blomqvist *et al.*, 1993). This indicates that the growth rate in different subclones of the sarcomas in this study seems to be quite constant. One study in breast cancer reported relatively stable SPF values in primary tumours and metastases (Feichter *et al.*, 1989), whereas two studies in ovarian carcinoma both indicated considerable variation in SPF between different samples from the primary tumour, and between primary tumours and metastases in the same patient (Kaerne *et al.*, 1994; Kallioniemi 1988). Interestingly, the heterogeneity of SPF both in breast and ovarian cancer was reported to be larger in non-diploid tumours (Feichter *et al.*, 1989; Kaerne *et al.*, 1994).

Secondly, there might be a correlation between CLF or $T_S$ and SPF. There is little data available on this issue. The slope of the regression line between log ($T_2$) and log(SPF) was, however, close to the theoretical value of $-1$, which should not be the case if either $T_S$ or CLF were strongly correlated to SPF (for elaboration see Materials and methods).

Thirdly, patients with measurable lung metastases might be a non-random subset of sarcoma patients in general; in our department only 25 out of more than 200 sarcoma patients treated during the period of the study fulfilled the inclusion criteria.

An unexpected finding was that the correlation between SPF and clinical growth of metastases was closer in diploid than in non-diploid tumours. In fact, one would expect the opposite owing to the inevitable contamination of normal cells in the SPF estimate in diploid tumours. The difference might naturally be caused by chance in this relatively small patient sample. The regression equations between $T_2$ and SPF were, however, almost identical in diploid and non-diploid tumours, but the non-diploid tumours showed much larger variability. This may indicate that other factors responsible for $T_2$ (i.e. CLF and $T_S$) than SPF might be more important determinants of $T_2$ in non-diploid tumours. Two previous studies, one of 100 colorectal cancer cases and the other of 47 patients with head and neck carcinomas, have demonstrated significantly longer $T_S$ times in non-diploid than in diploid tumours indicating systematic differences in $T_S$ between diploid and non-diploid tumours (Begg *et al.*, 1990; Rew *et al.*, 1991). Interestingly, in a recent study of the patient material from which the present patients were recruited SPF was a strong predictor of metastatic development and survival in diploid tumours only (Huuhtanen *et al.*, 1995).

The estimated regression equation can be approximately reformulated in the form $T_2 = 300/SPF$, enabling a simple method of estimating the expected tumour doubling time from SPF in individual cases. In non-diploid tumours, however, this estimate is relatively inexact, since SPF explained only about 10% of the variability (variance) in $T_2$, whereas SPF explained about 60% of the variation in diploid tumours.

**Acknowledgements**

The study was supported by King Gustaf V's Jubilee foundation, by a grant donated by Zeneca Pharma to the foundation for Finnish Cancer Institute and by the Clinical Research Institute of the Helsinki University Hospital.

### References

BEGG AC., MOONEN L, HOFLAND I, DESSING M AND BARTELINK H. (1988). Human tumour cell kinetics using a monoclonal antibody against iododeoxyuridine: intratumour sampling variations [published erratum appears in *Radiother. Oncol.*, **15**, 215]. *Radiother. Oncol.*, **11**, 337-347.

BEGG AC, HOFLAND I, MOONEN L, BARTELINK H, SCHRAUB S, BONTEMPS P, LE FUR R, VAN DEN BOGAERT W, CASPERS R, VAN GLABBEKE M AND HORIOT JC. (1990). The predictive value of all kinetic measurements in a European trial of accelerated fractionation in advanced head and neck tumors: an interim report. *Int. J. Radiat. Oncol. Biol. Phys.*, **19**, 1449-1453.

BLOMQVIST C, WIKLUND T, TARKKANEN M, ELOMAA I AND VIROLAINEN M. (1993). Measurement of growth rate of lung metastases in 21 patients with bone or soft-tissue sarcoma. *Br. J. Cancer*, **68**, 414-417.

CHARBIT A, MALAISE EP AND TUBIANA M. (1971). Relation between the pathological nature and the growth rate of human tumors. *Eur. J. Cancer*, **7**, 307-315.

FEICHTER GE, KAUFMANN M, MÜLLER A, HAAG D, ECKHARDT R AND GOERTTLER K. (1989). DNA index and cell cycle analysis of primary breast cancer and synchronous axillary lymph node metastases. *Breast Cancer Res. Treat.*, **13**, 17-22.

FORSTER G, COOKE TG, COOKE LD, STANTON PD, BOWIE G AND STELL PM. (1992). Tumour growth rates in squamous carcinoma of the head and neck measured by *in vivo* bromodeoxyuridine incorporation and flow cytometry. *Br. J. Cancer*, **65**, 698-702.

HALL PA AND LEVISON DA. (1992). Review: Assessment of cell proliferation in histological material. *J. Clin. Pathol.*, **43**, 184-192.

HEIDEN T, STRANG P, STENDAHL U AND TRIBUKAIT B. (1990). The reproducibility of flow cytometric analyses in human tumors. Methodological aspects. *Anticancer Res.*, **10**, 49-54.

HEIDEN T, WANG N AND TRIBUKAIT B. (1991). An improved Hedley method for preparation of paraffin-embedded tissues for flow-cytometric analysis of ploidy and S-phase. *Cytometry*, **12**, 614-621.

HUUHTANEN R, WIKLUND T, BLOMQVIST C, VIROLAINEN M, YI P AND TRIBUKAIT B. (1995). High S-phase fraction is an adverse prognostic sign in diploid soft-tissue sarcomas. *Submitted*,

JOSEPH WL, MORTON DL AND ADKINS PC. (1971). Prognostic significance of tumor doubling time in evaluating operability in pulmonary metastatic disease. *J. Thoracic Cardiovascular Surg.*, **61**, 23-32.

KAERNE J, TROPÉ CG, KRISTENSEN GB AND PETTERSEN EO. (1994). Flow cytometric DNA ploidy and S-phase heterogeneity in advanced ovarian carcinoma. *Cancer*, **73**, 1870-1877.

KALLIONIEMI O-P. (1988). Comparison of fresh and paraffin-embedded tissue as starting material for DNA flow cytometry and evaluation of intratumour heterogeneity. *Cytometry*, **9**, 164-169.

MALAISE EP, CHAVAUDRA N AND TUBIANA M. (1973). The relationship between growth rate, labelling index and histological type of human solid tumours. *Eur. J. Cancer*, **9**, 305-312.

MATTSON K AND HOLSTI LR. (1980). Prognostic value of doubling time in lung cancer. *Strahlenterapie*, **156**, 623-636.

RAZA A, PREISLER HD, DAY R, YASIN Z, WHITE M, LYKINS J, KUKLA C, BARCOS M, BENNETT J, BROWMAN G, GOLDBERG J, GRÜNWALD H, LARSON R, VARDIMAN J AND VOGLER R. (1990). Direct relationship between remission duration in acute myeloid leukemia and cell cycle kinetics: a leukemia intergroup study. *Blood*, **76**, 2191-2197.

REW DA, WILSON GD, TAYLOR I AND WEAVER PC. (1991). Proliferation characteristics of human colorectal carcinomas measured *in vivo*. *Br. J. Surg.*, **78**, 60-66.

REW DA, CAMPBELL ID, TAYLOR I AND WILSON GD. (1992). Proliferation indices of invasive breast carcinomas after *in vivo* 5-bromo-2'-deoxyuridine labelling: a flow cytometric study of 75 tumours. *Br. J. Surg.*, **79**, 335-339.

RICCARDI A, DANOVA M, WILSON G, UCCI G, DORMER P, MAZZINI G, BRUGNATELLI S, GIRINO M, MCNALLY NJ AND ASCARI E. (1988). Cell kinetics in human malignancies studied with *in vivo* administration of bromodeoxyuridine and flow cytometry [published erratum appears in *Cancer Res*, **53**, 4119]. *Cancer Res.*, **48**, 6238-6245.

SAKUMA J. (1980). Cell kinetics of human squamous cell carcinomas in the oral cavity. *Bull. Tokyo Med. Dent. Univ.*, **27**, 43-54.

SILVESTRINI R, SANFILIPPO O AND TEDESCO G. (1974). Kinetics of human mammary carcinomas and their correlation with the cancer and the host characteristics. *Cancer*, **34**, 1252-1258.

SPRATT JS AND SPRATT TL. (1964). Rates of growth of pulmonary metastases and host survival. *Ann. Surg.*, **159**, 161-171.

STEEL GG. (1967). Cell loss as a factor in the growth rate of human tumours. *Eur. J. Cancer*, **3**, 381-387.

WILSON GD, MCNALLY NG, DISCHE S, SAUNDERS MI, DES RC, LEWIS AA AND BENNETT MH. (1988). Measurement of cell kinetics in human tumours *in vivo* using bromodeoxyuridine incorporation and flow cytometry. *Br. J. Cancer*, **58**, 423-431.